# The Mechanical Properties and Degradation Behavior of 3D-Printed Cellulose Nanofiber/Polylactic Acid Composites

**DOI:** 10.3390/ma16186197

**Published:** 2023-09-13

**Authors:** Zhongsen Zhang, Bingyan Cao, Ning Jiang

**Affiliations:** 1 School of Aerospace Engineering and Applied Mechanics, Tongji University, Shanghai 200092, China; 2 School of Transportation and Vehicle Engineering, Shandong University of Technology, Zibo 255049, China

**Keywords:** polylactic acid, cellulose nanofiber, composites, degradation behavior, mechanical properties, 3D printing

## Abstract

Polylactic acid (PLA) has been widely used in many fields because of its good biodegradability, biocompatibility, and renewability. This work studied the degradation behavior and mechanical properties of cellulose nanofiber (CNF)/PLA composites. In vitro degradation experiments of 3D-printed samples were conducted at elevated temperatures, and the degradation characteristics were evaluated by mechanical tests, gel permeation chromatography (GPC), differential scanning calorimetric (DSC), and scanning electron microscope (SEM). The results indicated that the addition of CNF (0.5 wt%) accelerated the degradation rate of PLA. The decreases in number average molecular weight ($M_{n}$) and weight average molecular weight $\left(M_{w}\right)$ of composites were 7.96% and 4.91% higher than that of neat PLA, respectively. Furthermore, the tensile modulus of composites was 18.4% higher than that of neat PLA, while the strength was 7.4% lower due to poor interfacial bonding between CNF and PLA. A mapping relationship between accelerated and normal degradation showed that the degradation experienced during 60 days at 37 °C was equivalent to that undergone during 14 days at 50 °C; this was achieved by examining the alteration in $M_{n}$. Moreover, the degradation process caused a notable deformation in the samples due to residual stress generated during the 3D printing process. This study provided valuable insights for investigating the in vitro degradation behavior of 3D-printed products.

## 1. Introduction

Poly(lactic acid) (PLA), which has the advantages of good biodegradability, biocompatibility, renewability, and easy processing, is widely used in biomedical engineering fields, such as bone nails, surgical sutures, and vascular stents [1,2,3,4,5]. It can be degraded by hydrolysis of ester bonds in aqueous environments such as body fluids, which can be divided into four steps: polymer swelling; break of ester bonds; diffusion of soluble degradation products (lactic acid and oligomer); and extinction of lactic acid by metabolism. As a biodegradable material, the degradation behavior of PLA is the key index for measuring its performance, and it is very important when seeking to realize controllable degradation. Moreover, when compared to common metal materials, PLA exhibits inferior mechanical properties [6]. To meet the needs of different applications, blending and copolymerization techniques are often used to prepare PLA composites in order to regulate the degradation rate and enhance the mechanical properties of the materials.

The regulation of the degradation rate is usually accompanied by changes in mechanical properties. Toncheva′s research showed that the hydrolysis rate of PLA grafted by PEG was increased compared with neat PLA [7]. It has also been proved that simple blending of PEG and PLA can accelerate the degradation of materials, which is attributed to the hydrophilicity of PEG. However, the addition of PEG often leads to the reduction of mechanical strength [8,9]. Feng et al. blended l-lactic (L-LA) with PLA, which increased the amorphous region by hindering the crystallization of PLA. This accelerated the hydrolysis of PLA but also decreased the tensile strength and modulus of the composites [10]. Starch exhibits a similar promoting effect as it not only increases the hydrophilicity of the material but also destroys the crystal structure of PLA. This disruption makes it easier for water molecules to permeate, thus increasing the hydrolysis rate [11,12,13,14], which leads to a decrease in strength and modulus. There are also cases of delaying the degradation of PLA. The addition of hydroxyapatite (HA) to PLA was performed to neutralize the acidic environment and weaken the autocatalytic effect of the degradation of PLA. This blending process not only decreased the hydrolysis of the composites but also increased their mechanical strength [15,16]. In the carbon nanotube (CNTs)/PLA composite sutures, the addition of CNTs enhanced their crystallinity and orientation when compared with neat PLA, which prolonged the degradation time of sutures and improved their strength [3].

Nanocellulose is a kind of natural material with various names depending on its preparation methods and structural dimensions, mainly including cellulose nanofiber (CNF), cellulose nanocrystal (CNC), and bacterial nanocellulose (BNC) [17]. Nanocellulose has high strength, high modulus, good biocompatibility, renewability, and degradability. It serves as an excellent reinforcing material to strengthen the PLA matrix [18,19]. More importantly, nanocellulose has superior biocompatibility and bioactivity when compared with other reinforcing phases (such as CNTs) [20]. As a hydrophilic material and nano-filler, nanocellulose helps to improve the moisture absorption capacity [19,21]. There are many factors affecting the degradation of nanocellulose/PLA composites. The hygroscopic ability of nanocellulose can promote the hydrolysis of the PLA matrix [22,23,24]. However, it also acts as a nucleating agent, leading to increased crystallinity, which in turn delays the hydrolysis of PLA [25,26]. In addition, the interface defects of composites can provide space for water molecules, promoting the degradation of the PLA matrix.

Compared to traditional manufacturing methods, 3D printing offers limitless design possibilities, enables the creation of complex shapes, and minimizes material waste. It is particularly suitable for the production of medical implants such as vascular stents, pancreaticojejunostomy devices, and bone tissue engineering scaffolds [4,27,28,29]. However, the durability of 3D-printed parts has not been thoroughly investigated or disclosed. Moreover, the fused deposition molding (FDM) process, which is widely employed in 3D printing, often produces substantial thermal residual stress in the printed parts due to repeated heating and cooling cycles [30,31]. This residual stress can be relieved in a humid environment, leading to structural deformation and ultimately affecting the usability and functionality of the component. Additionally, lots of defects arise during the FDM process [31], which would also affect the degradation behavior of the 3D-printed parts. Therefore, conducting research on the durability of 3D-printed components in a hygrothermal environment is important for expanding their potential applications.

In this study, CNF-reinforced PLA nanocomposites were first prepared using the FDM 3D printing technique. In vitro accelerated degradation experiments were then conducted under simulated body fluid conditions at elevated temperatures to assess the degradation behavior of 3D-printed CNF/PLA composites for biomedical purposes. Mechanical testing, gel permeation chromatography (GPC), differential scanning calorimetry (DSC), and scanning electron microscopy (SEM) were utilized to evaluate the impact of CNF addition on the degradation behavior of PLA. The correlation relationship between accelerated degradation and normal degradation was revealed by a degradation kinetic model. Furthermore, significant deformation in the 3D-printed parts was observed during the degradation process, and the deformation mechanism was specifically analyzed. The findings of this study are expected to provide valuable insights for investigating the in vitro degradation behavior of 3D-printed products.

## 2. Materials and Methods

### 2.1. Materials

The PLA powder used in this study was supplied by Huachuang plastic Co., Ltd., Kunshan, China, with a particle size of 250 meshes, which was prepared by cryogenically grinding of PLA pellets (4032D, NatureWorks LLC, Lake Ariel, PA, USA).

The CNF aqueous suspension was supplied by Jinjiahao Green Nanomaterials Co., Ltd., Quzhou, China. The phosphate buffered saline (PBS, 0.01 M, pH = 7.4) used as simulated body fluid in this research was supplied by Hewei Pharmaceutical Technology Co., Ltd., Hefei, China.

### 2.2. Preparation of CNF/PLA Composites

**Figure 1:**
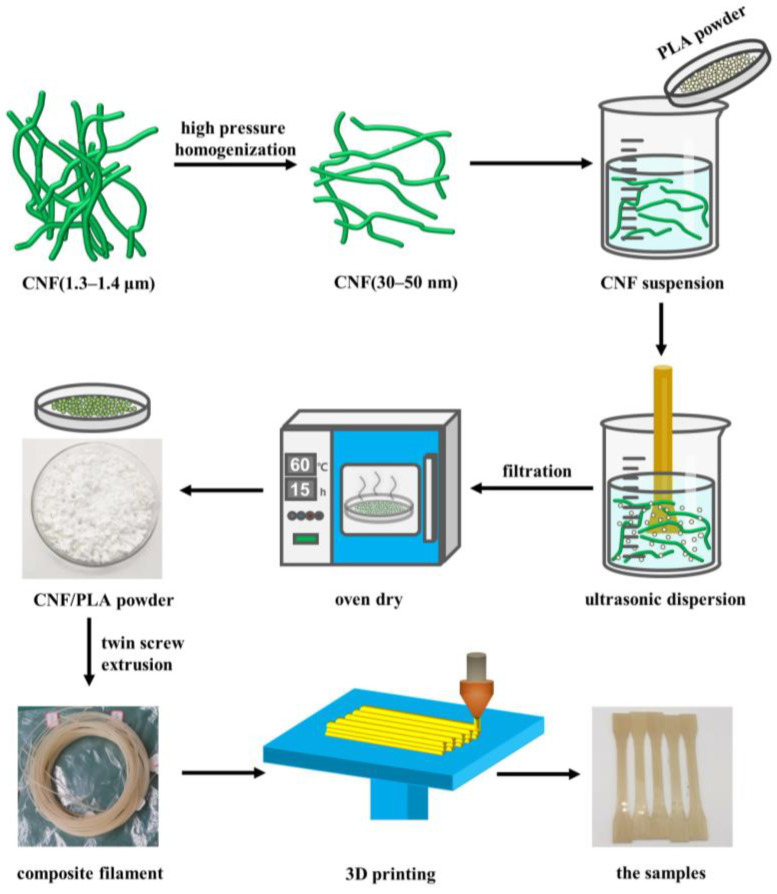
The preparation process of samples.

**Figure 2:**
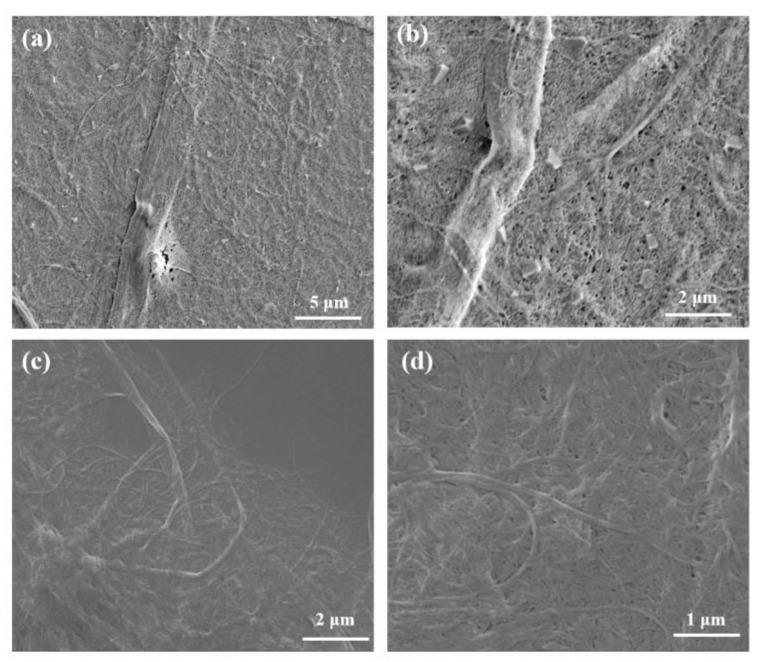
SEM images of cellulose nanofibers: (**a**,**b**) before homogenization and (**c**,**d**) after homogenization.

The sample preparation process is depicted in Figure 1. To minimize the agglomeration of CNFs and achieve a more uniform blending of PLA and CNF, the PLA micro powders were directly mixed with the CNF aqueous suspension. Initially, the CNF aqueous suspension underwent homogenization (800 bar, 10 cycles) using a high-pressure homogenizer (FB-110 × 1, Litu Ultra-high Pressure Equipment Co., Ltd., Shanghai, China) to obtain CNFs with a smaller diameter and a larger length-diameter ratio, as referenced in [32]. After the high-pressure homogenization, the CNF diameter was reduced from around 1.5 μm to 30~50 nm, as depicted in Figure 2. A total of 150 g of PLA powder was weighed, and the CNF aqueous suspension was measured accordingly to attain a CNF content of 0.5 wt%. The PLA powders were then blended with the CNF aqueous suspension and subjected to ultrasonic dispersion for 30 min using an ultrasonic processor (FS-600N, Bioanassonic Instrument Co., Ltd., Shanghai, China) with a power of 420 W. Excess water was eliminated through positive pressure filtration, and the mixture was dried at 60 °C in an oven for approximately 15 h to obtain CNF/PLA powders.

**Figure 3:**
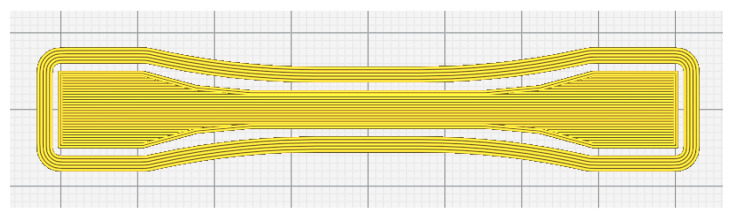
The infill pattern for 3D printing of the specimens.

Filament extrusion was performed using a conical twin-screw extruder (WLG10AG, Xinshuo Precision Machinery Co., Ltd., Shanghai China). Each time, 7.5 g of CNF/PLA composite powders were introduced into the chamber and extruded after 10 min of cyclic blending. During the extrusion process, the temperature of the upper and lower chamber plates was maintained at 175 °C, the barrel temperature was set to 165 °C, and the filament diameter was controlled at 1.75 mm, as referenced in [33]. Dumbbell-shaped specimens (based on ISO 527-2 [34]) were utilized and prepared using a FDM 3D printer (New X1, Infinity) according to the processing parameters listed in Table 1. The infill pattern used for 3D printing the specimens is a straight line along the length direction, as illustrated in Figure 3.

### 2.3. In Vitro Degradation

PLA undergoes degradation through hydrolysis of ester bonds in aqueous environments at 37 °C, such as body fluids. The hydrolysis and breaking of the molecular chains result in the generation of carboxyl groups, which in turn catalyze the degradation process of PLA [36]. As a result, the degradation products tend to accumulate internally, leading to a higher concentration of carboxyl groups inside when compared to the surface. This causes the degradation rate of the internal parts of PLA samples to be faster than that of the surface. With continued degradation, the accumulation of carboxyl groups within the material further exacerbates the difference between the internal and external degradation rates. This phenomenon is referred to as the autocatalytic effect [37,38].

Significant degradation can be observed only at high temperatures or over long periods of time. In order to assess the long-term performance of the samples within a short period, three degradation temperatures (37 °C, 50 °C, and 60 °C) were selected. 37 °C is the human body temperature, which is considered as the normal ambient temperature for degradation. The glass transition temperature ($T_{g}$) of PLA is approximately 60 °C. If the degradation is accelerated beyond this temperature, it becomes challenging to measure the change in hydrolysis rate with temperature [39]. Therefore, 60 °C was selected as the maximum temperature. Additionally, 50 °C was selected as it falls between the two previously mentioned values. These three temperatures were chosen to establish the correlation between hydrolysis rate and temperature, allowing for the prediction of hydrolysis rate at any temperature below $T_{g}$. The normal degradation experiment and accelerated degradation experiment were conducted simultaneously, with a focus on analyzing the degradation behavior at 37 °C and 50 °C. Before initiating the degradation experiment, the samples were dried and weighed. The weight measurement was performed three times, and the average value was recorded as $m_{0}$. During degradation, the samples were fully immersed in PBS, ensuring that the volume of PBS (mL) exceeded the sample weight (g) by at least 30 mL/g, by ISO 15814 standard [40]. The samples, along with the PBS, were placed in glass bottles and submerged in constant temperature sinks (DK-500, Jinghong Experimental Equipment Co., Ltd., Shanghai, China). The culture solution was changed every week. At specific time points (0, 7, and 14 days), the samples were removed from the bottles for characterization purposes.

### 2.4. Characterization

Molecular weight analysis. The number average molecular weight ($M_{n}$) and weight average molecular weight ($M_{w}$) and their distribution ($M_{w}/M_{n}$) of the samples were determined by gel permeation chromatography (GPC) system (ACQUITY APC, Waters Technology Co., Ltd., Taunton, MA, USA). After degradation, the samples were rinsed with deionized water and then dried. Subsequently, the samples were dissolved in chloroform, and the PLA and CNF components were separated by filtration. Polystyrene was employed as the standard material.

Differential scanning calorimetric (DSC) analysis. The thermal properties of the samples were determined using differential scanning calorimetry (DSC) (Q20, TA Instruments, New Castle, DE, USA), including the glass transition temperature ($T_{g}$), cold crystallization temperature ($T_{cc}$), melting temperature ($T_{m}$), cold crystallization enthalpy ($\Delta H_{cc}$), and melting enthalpy ($\Delta H_{mm}$). All the samples were heated from 20 °C to 200 °C at the heating rate of 20 °C/min, then retained heat for 5 min. Then, they were cooled to 20 °C at the cooling rate of 20 °C/min, then kept at this heat for 5 min, and heated to 200 °C again at the heating rate of 10 °C/min. The flow rate of nitrogen gas was 50 mL/min. The crystallinity ($\chi_{c}$) of degraded samples was calculated using the first heating curve, and it was calculated according to Equation (1). Due to the low CNF content (only 0.5 wt%), the PLA mass fraction ($\chi_{PLA}$) was taken as 1. The melting enthalpy of 100% crystalline PLA ($\Delta H_{0}$) was taken from the literature as 93.1 J/g [41].

(1) $$\chi_{c}\left(\%\right)=\frac{\Delta H_{m}-\Delta H_{cc}}{\Delta H_{0}\times \chi_{PLA}}\times 100\%$$

Mechanical performance analysis. The dimensions of the specimens for the tensile test were 80 × 10 × 2 mm^3^. Their shape was in accordance with the 1BA specimen of the ISO 527 standard. The tensile strength and tensile modulus of the specimens were determined using the electronic universal testing machine (Model 100ST, Tinius Olsen Corporation, Horsham, PA, USA), with a loading velocity of 1 mm/min. Five specimens were tested for each group, and the average value was taken as the final result. The tests were conducted immediately after removing the specimens from the aqueous environment. Before the mechanical performance tests, the specimens were immersed in distilled water at 37 °C for 60 min to meet the state adjustment requirements of ISO 15814 standard. To ensure stability during testing, the clamping segments were thoroughly dried before the test commenced.

Morphological analysis. A field emission environmental scanning electron microscope (Quanta 200 FEG, FEI Corporation, Hillsboro, OR, USA) was used to observe the micromorphology of the cross sections of samples before and after degradation at 20 kV. The samples were coated with gold before observation.

## 3. Results and Discussion

### 3.1. Molecular Weight Analysis

**Figure 4:**
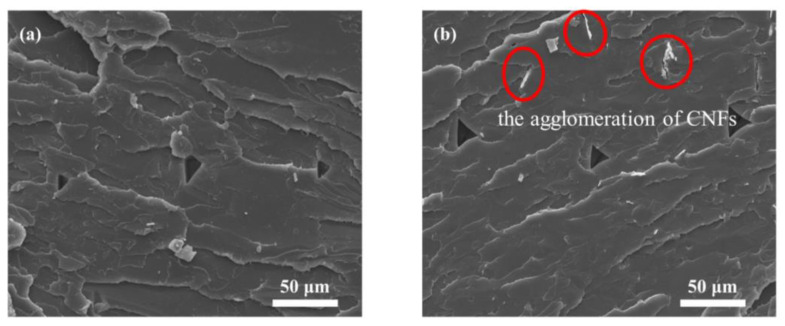
The cross-sectional morphologies of (**a**) neat PLA and (**b**) CNF/PLA composites before degradation. (Red circles indicate the agglomeration of CNFs.)

The degree of degradation of biodegradable polymers can be most significantly indicated by the change in molecular weight [2,42]. Table 2 presents the values of $\;M_{n}$, $M_{w}$, and $M_{w}/M_{n}$ for both CNF/PLA composites and neat PLA samples after accelerated degradation at 50 °C. Prior to degradation, the $M_{n}$, $M_{w}$, and $M_{w}/M_{n}$ of neat PLA in the samples were 3.7 × 10^4^, 6.7 × 10^4^, 1.82, respectively. The molecular weight of the samples decreased following degradation, indicating the hydrolysis and breakage of high molecular chains into lower molecular weight products. After degradation, the $M_{n}$ and $M_{w}$ of neat PLA samples decreased by 65.6% and 61.1%, respectively. It is assumed that the molecular weights of CNF/PLA composite remain similar to neat PLA before degradation [35]. The $M_{n}$ and $M_{w}$ of CNF/PLA samples decreased by 70.8% and 64.1%, respectively, compared to neat PLA. It is evident that under the same degradation conditions, the degree of degradation for CNF/PLA composite samples was higher. This can primarily be attributed to the hydrophilic nature of CNF and the enhanced moisture absorption rate exhibited by the composite samples compared to neat PLA samples. The cross-sectional morphologies of neat PLA and CNF/PLA composites before degradation are shown in Figure 4. It is noticeable that numerous aggregated CNF flakes are embedded into the PLA matrix, with sizes ranging in the dozens of micrometers. This suggests that the processing using cyclic twin-screw extrusion could hardly disperse the CNF networks into nanoscale reinforcement during the blending process due to the strong hydrogen bonding among CNFs. The poor interfacial compatibility between CNF and PLA due to the agglomeration of CNFs resulted in more internal defects in the composite samples, facilitating the permeation of water molecules and thus accelerating degradation. The polydispersity index ($M_{w}/M_{n}$) is typically used to quantify the polydispersity of polymers. During degradation, the molecular weight decreased, leading to an acceleration in the hydrolysis rate of molecular chains. Consequently, the generation of more degradation products with lower molecular weight results in wider molecular weight distribution.

The first-order reaction kinetic equation is used to represent the change in the number average molecular weight of PLA in the samples over time [38,43], i.e., Equation (2)

(2) $$lnM_{n}=A-k\cdot t$$

In Equation (2), A (the logarithm of the initial $M_{n}$) is a constant, which is 10.508. k is the degradation rate and t is the time. Considering the influence of $T_{g}$ (about 60 °C), the Vogel–Tammann–Fulcher equation is used to describe the degradation rate instead of the Arrhenius equation here [39], i.e., Equation (3)

(3) $$k=k_{0}\cdot e^{\frac{-E_{a}}{R}\cdot \frac{1}{T-T_{s}}}$$

where $k_{0}$ is a constant, $E_{a}$ is the activation energy, R is the gas constant, T is the temperature, and $T_{s}$ is the reference temperature when the conformational entropy of the polymer molecular chains is close to 0, which is generally about 50 °C lower than $T_{g}$ [44], and it is taken 10 °C in this study. Combine Equations (2) and (3) to obtain Equation (4)

(4) $$ln(A-lnM_{n})=lnk_{0}+lnt-\frac{-E_{a}}{R}\cdot \frac{1}{T-T_{s}}$$

**Figure 5:**
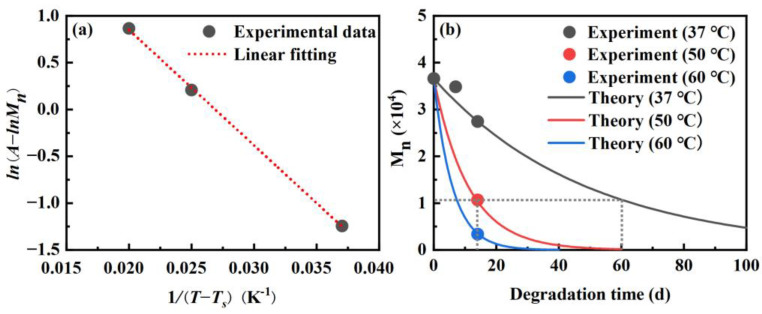
(**a**) The linear fitting and (**b**) the theoretical curves and experimental data about the relationship between $M_{n}\;$ and degradation time at different temperatures.

$ln(A-lnM_{n})$ is taken as the dependent variable and $1/T-T_{s}$ is taken as the independent variable. The experimental data after 14 days of degradation at different temperatures (37 °C, 50 °C and 60 °C) are fitted linearly, as shown in Figure 5a. From this fit, we can obtain that $E_{a}/R=123.27\;K$, $k_{0}=1.9632{\;\mathrm{day}}^{-1}$.

Equation (4) can also be transformed into the following form:

(5) $$M_{n}=e^{A-k_{0}\cdot t\cdot e^{\frac{-E_{a}}{R}\cdot \frac{1}{T-T_{s}}}}$$

The known values A,$\;E_{a}/R$, and $k_{0}$ were incorporated into Equation (5) to generate function curves illustrating the degradation of PLA in the samples at various temperatures, as depicted in Figure 5b. It is evident that the theoretical data are in excellent agreement with the experimental data. Due to the low initial $M_{n}$ value of 3.7 × 10^4^ for the PLA utilized in this study, the degradation rate is relatively rapid. According to the theoretical curves, after 100 days of degradation at 37 °C, $M_{n}$ has decreased to approximately 5000 Da, representing an 86.1% reduction. At this stage, the samples have lost their mechanical properties. Additionally, it can be inferred that the degradation undergone during 60 days at 37 °C is equivalent to that experienced during 14 days at 50 °C by examining the alteration in $M_{n}$.

### 3.2. Thermal Properties Analysis

**Figure 6:**
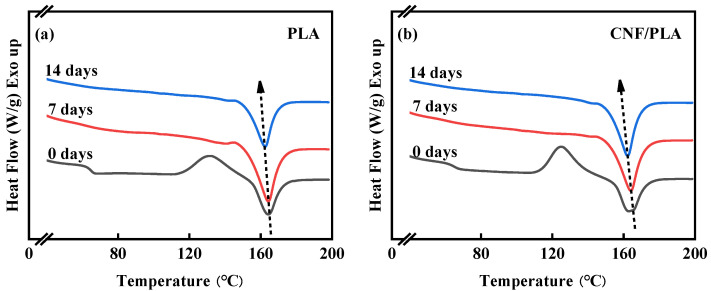
The first heating DSC curves of degraded samples at 0, 7, and 14 days, (**a**) neat PLA and (**b**) CNF/PLA composites.

The first heating DSC curves of neat PLA and CNF/PLA composite samples before and after degradation at 50 °C are illustrated in Figure 6, and related values are shown in Table 3. Before degradation, the neat PLA and CNF/PLA composite present similar $T_{g}$ and $T_{m}$, which was also reported in reference [45]. Nevertheless, there is a slight decrease in the *T_cc_* with the addition of CNF. The presenting of CNF as nucleating agents for the crystallization of PLA reduces the energy required to initiate the crystallization process.

It is evident that the $T_{m}$ of the samples shifted towards lower temperatures after degradation. This can be attributed to the degradation-induced breakage of molecular chains, resulting in more disturbed or less prefect crystals, ultimately leading to a reduction in $T_{m}$ [46]. Figure 6 demonstrates the disappearance of the glass transition and cold crystallization peaks in both neat PLA and CNF/PLA composite samples following accelerated degradation at 50 °C. Similar phenomena have also been reported in previous works [15,47]. Calculated from Equation (1), the crystallinity of the degraded samples significantly increased compared to their pre-degradation state. This can be attributed to the initial hydrolysis of the amorphous region in PLA, resulting in a higher proportion of the crystalline region. Furthermore, enhanced molecular segmental mobility facilitated the more orderly arrangement of chains [15,47].

**Figure 7:**
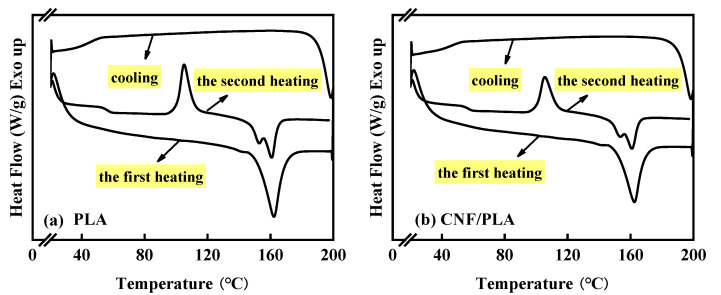
The first and second heating DSC curves of degraded samples at 50 °C for 14 days, (**a**) neat PLA and (**b**) CNF/PLA composites.

Figure 7 displays the first and second heating curves of pure PLA and CNF/PLA composite samples after accelerated degradation for 14 days at 50 °C. The first heating curve reflects the thermal properties of the samples following their thermal history (i.e., in vitro degradation), while the second heating curve reveals the true properties of the samples after eliminating the thermal history. Unlike the first heating curves, distinct glass transitions and cold crystallization peaks can be observed during the second heating process. The degradation process substantially increased the crystallinity of the samples, impeding the movement of chain segments in the amorphous region and making the detection of $T_{g}$ challenging on the first heating curves. With the cleavage of molecular chains coupled with the plasticizing effect of water, molecular segmental mobility was enhanced, facilitating chain arrangement and resulting in easier crystallization and a more perfect crystal structure, thereby eliminating the cold crystallization peaks. The rapid cooling rate following the first heating led to an imperfect crystal structure. This is evident in Figure 7, where the crystallinity after the second heating is considerably lower than that after the first heating. Therefore, a significant glass transition and cold crystallization peak occur during the second heating process.

### 3.3. Morphological Analysis

**Figure 8:**
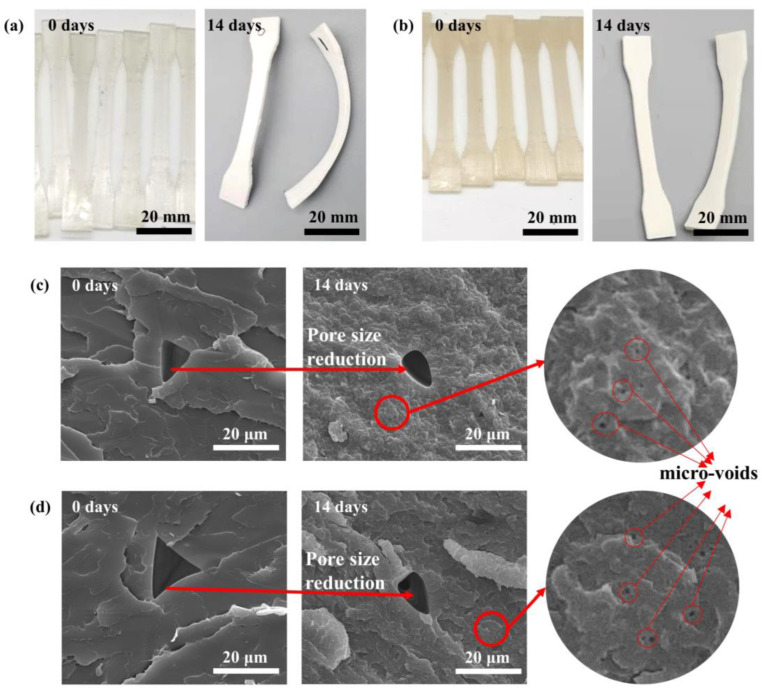
The macroscopic and microscopic morphologies of (**a**,**c**) neat PLA and (**b**,**d**) CNF/PLA composite before and after degradation at 50 °C.

The macroscopic and microscopic morphology of neat PLA and CNF/PLA composite samples before and after degradation at 50 °C for 14 days are presented in Figure 8. Before degradation, the neat PLA samples exhibited transparency, while the CNF/PLA composite samples displayed a brownish-yellow color, indicating reduced transparency. It was previously mentioned that the crystallinity of the samples significantly increased after degradation. This increase in crystallinity led to more severe scattering due to the different refractive indexes of light in the crystalline and amorphous regions. Consequently, the degraded samples shown in Figure 8a,b appeared as a non-transparent milky white color. Moreover, Figure 8a,b clearly show that the samples experienced substantial deformation after 14 days of degradation, which will be discussed in detail later.

The SEM micrographs (Figure 8c,d) reveal that the cross sections of both neat PLA and CNF/PLA composite samples exhibited rougher surfaces compared to their pre-degradation state. The tensile fracture morphology confirms that 3D-printed PLA exhibits a typical brittle fracture, which is further confirmed by the measured value of the elongation at break. The addition of CNF did not cause a significant alteration in the fracture behavior of the composites, due to extremely low CNF content. Additionally, a significant number of micro-voids were observed within the samples. This occurrence is attributed to the autocatalytic effect of PLA degradation. Since there were unavoidable pores in the 3D printed samples [48], the infiltration of water molecules resulted in swelling of the PLA and a reduction in the size of internal pores.

### 3.4. Deformation Analysis

**Figure 9:**
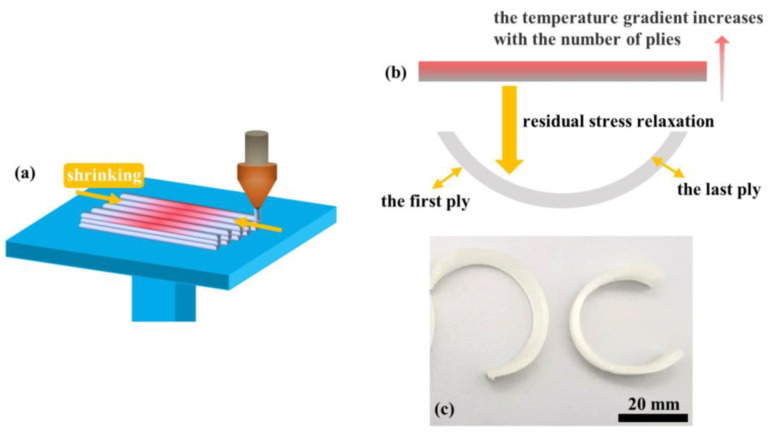
Schematic drawings of (**a**) structural shrinking during 3D printing and (**b**) the relaxation process of residual stress. (**c**) The deformed specimens after degradation.

As mentioned previously, the specimens in the accelerated degradation experiments underwent significant deformation, which can be attributed to the presence of residual stress in the 3D-printed specimens. Figure 9a illustrates the 3D printing process where the material is deposited on the printing bed after undergoing melt extrusion. The cooling fan positioned above the nozzle assists in the cooling and solidification processes, leading to rapid material cooling and shrinkage. However, due to bonding with the previous layer, contraction is prevented, resulting in internal stress within the material. In this study, the hot bed was maintained at a temperature close to $T_{g}$ (60 °C) during the printing process, which effectively reduced internal stress in the plies near the bottom. As the number of printed plies increased, the temperature difference between melting and cooling also increased, leading to higher internal stress in plies farther from the bottom. During degradation, the specimens were subjected to a heated state, where water acted as a plasticizer for the PLA, enhancing the movement of its molecular chain segments and releasing internal stress, as shown in Figure 9b. Higher temperatures in the accelerated degradation process resulted in an expedited release of internal stress. The specimens experienced free deformation in the solution, with greater residual stress near the last ply. Consequently, the specimens exhibited a “C” shape from the bending of the first ply to the last ply.

### 3.5. Mechanical Performance Analysis

**Figure 10:**
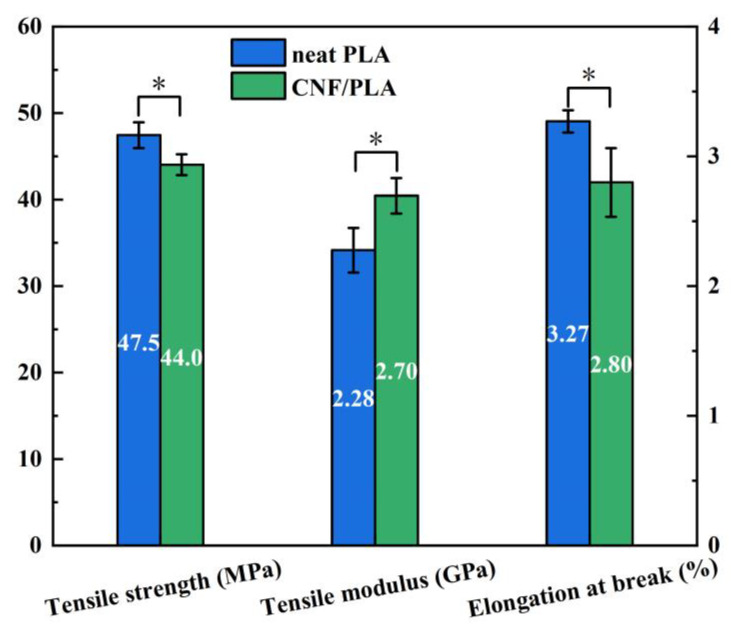
Comparison of tensile properties between neat PLA and CNF/PLA. (* indicates a statistically significant difference between the two groups at the 0.05 level).

The tensile strength of CNF/PLA composites was 44.0 MPa, which was 7.4% lower than that of PLA. However, CNF failed to act as a reinforcement and instead decreased the material’s tensile strength. The reason for this is the incompatibility between hydrophilic CNF and hydrophobic PLA. This leads to weak interfacial bonding and an increased occurrence of material defects as shown in Figure 4. The presence of a low CNF content leads to greater performance degradation caused by internal defects compared to the strengthening effect of CNF, consequently resulting in decreased material strength. Furthermore, CNF has a high tendency to agglomerate, resulting in limited dispersion within the PLA matrix. The tensile modulus of the CNF/PLA composites showed an 18.4% improvement compared to PLA. This indicates that the elastic modulus is less sensitive to internal defects than tensile strength. The extremely high modulus of elasticity exhibited by CNF ultimately resulted in an overall enhancement of the tensile modulus in the CNF/PLA composites. Figure 10 also presents a reduction in elongation at break for CNF/PLA composites. This occurs because the flexible polymer matrix is being replaced by rigid CNF fillers, making the composite brittle [35].

**Figure 11:**
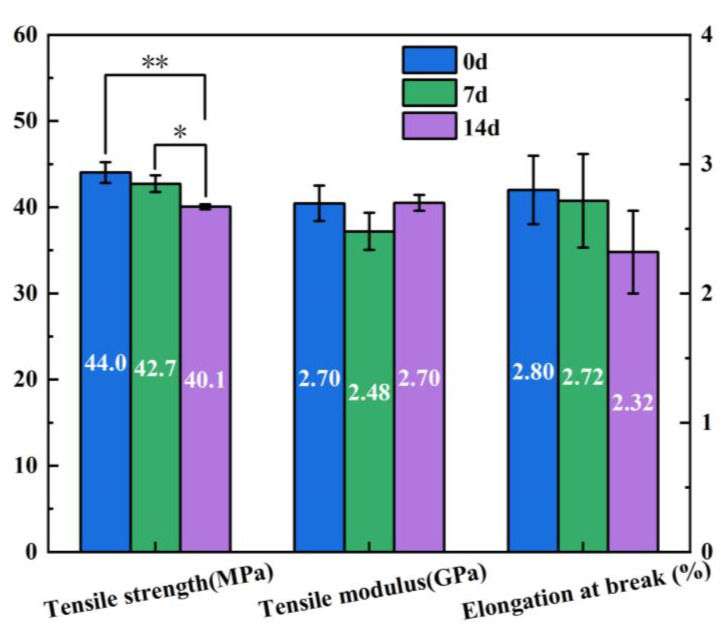
Changes in tensile properties of CNF/PLA composites after degradation at 37 °C (* indicates a statistically significant difference between the two groups at the 0.05 level, and ** indicates a significant difference at the 0.01 level).

In consideration of the extensive deformation of the accelerated degradation specimens, only the specimens subjected to normal degradation were tested. The specimens were retrieved at various time intervals for tensile testing, and the outcomes are illustrated in Figure 11. After 14 days of degradation at 37 °C, there was a significant difference in tensile strength. The 0.5 wt% CNF/PLA composites exhibited a decrease of 8.9% in tensile strength. This decline in tensile strength can primarily be attributed to the reduction in molecular weight, which subsequently affects the mechanical properties of the PLA matrix. Additionally, as described in Section 3.3, a substantial number of micro-voids emerged within the composites with degradation. This resulted in significant stress concentration around the voids, consequently diminishing their load capacities.

Although the average value of tensile modulus and elongation at break was reduced, the sensitivity analysis shows that no statistical differences were observed in tensile modulus and elongation at break due to the large data dispersivity. Section 3.1 clearly demonstrates that the poly-dispersity index of the PLA matrix has increased, indicating a broader molecular weight distribution that enhances the elastic modulus of the polymer. Additionally, an increase in crystallinity normally results in an increase in elastic modulus. Eventually, the composites exhibited a decline in both tensile strength and elongation at break throughout the degradation process. However, the tensile modulus initially decreased and then experienced a slight increment from day 7 to day 14. Moreover, the weakening of interfacial bonding between the CNF-reinforced phase and PLA matrix in the composites, as well as the elevated presence of defects after degradation, also contribute to the decline in mechanical properties.

## 4. Conclusions

In this study, the in vitro degradation behavior of 3D-printed biodegradable CNF/PLA composites were investigated. Mechanical testing, GPC, DSC, and SEM were utilized to evaluate the impact of CNF addition on the degradation behavior of PLA. The correlation relationship between accelerated degradation and normal degradation was revealed by a degradation kinetic model. Moreover, it was revealed that the degradation behavior of the FDM-printed CNF/PLA parts is distinct from those manufactured using traditional methods. The main differences lie in the presence of significant thermal residual stress and internal defects, which can result in notable deformation and accelerated moisture absorption during degradation. The following conclusions can be drawn:

(1) The addition of CNF increased the tensile modulus of the composites by 18.4% compared to neat PLA because of the high modulus of elasticity exhibited by CNF, while the strength of the composites was 7.4% lower due to poor interfacial bonding between CNF and PLA matrix. Over a degradation period of 14 days at 37 °C, the tensile strength of the CNF/PLA composites exhibited a decreased of 8.9%. This indicates a considerable susceptibility to the degradation conditions.
(2) The presence of CNF accelerated the degradation of the PLA. When subjected to accelerated degradation at 50 °C, the decreases of *M*_*n*_ and *M*_*w*_ of PLA matrix in the CNF/PLA composites were 7.96% and 4.91% higher than that of neat PLA.
(3) The mapping relationship between accelerated degradation and normal degradation implies that the degradation rate that occurs during 60 days at 37 °C is equivalent to that experienced during 14 days at 50 °C by examining the alteration in *M*_*n*_.
(4) The degraded samples exhibited improved crystallinity and crystallization capacity, while they appeared as a non-transparent milky white color with considerable amount of micro-voids generated inside the samples, and the addition of CNF caused a more pronounced change in the sample morphology.
(5) The degradation process caused a notable C-shape deformation in the 3D-printed parts due to residual stress generated during the 3D printing process. It is suggested to employ an appropriate post-treatment method to alleviate the residual stress in these 3D-printed parts prior to their application.

## Figures and Tables

**Table 1:** 3D printing parameters for PLA and CNF/PLA composites [35].

Parameter	Value
Nozzle diameter (mm)	0.4
Printing temperature (°C)	190
Bed temperature (°C)	60
Printing speed (mm/s)	50
Filling density	100%
Layer thickness (mm)	0.1
Printing flow	110%

**Table 2:** $M_{n}$, $M_{w}$, and $M_{w}/M_{n}$ of the samples before and after accelerated degradation at 50 °C.

Samples	Time (d)	$M_{n}\;\left(\mathrm{Da}\right)$	$M_{w}\;\left(\mathrm{Da}\right)$	$M_{w}/M_{n}$
neat PLA	0	3.7 × 10^4^	6.7 × 10^4^	1.82
neat PLA	14	1.3 × 10^4^	2.6 × 10^4^	2.06
CNF/PLA	14	1.1 × 10^4^	2.4 × 10^4^	2.24

**Table 3:** $T_{g}$, $T_{cc}$, $T_{m}$, $\Delta H_{m}$, $\Delta H_{cc}$, and $\chi_{c}$ of samples before and after accelerated degradation.

Samples	Time (d)	$T_{g}$ (℃)	$T_{cc}$ (℃)	$T_{m}$ (℃)	$\Delta H_{m}$ (J/g)	$\Delta H_{cc}$ (J/g)	$\chi_{c}$ (%)
neat PLA	0	64.75	131.69	164.27	22.00	15.83	6.63
neat PLA	7	--	--	164.00	22.36	0	24.01
neat PLA	14	--	--	162.27	22.04	0	23.67
CNF/PLA	0	64.81	127.41	165.31	24.90	21.45	3.71
CNF/PLA	7	--	--	164.26	23.63	0	25.29
CNF/PLA	14	--	--	162.43	22.40	0	24.06

## Data Availability

Data available on request due to restrictions of privacy.

## References

[1] Al-Shalawi F.D., Hanim M.A., Ariffin M.K.A., Kim C.L.S., Brabazon D., Calin R., Al-Osaimi M.O. (2023). Biodegradable synthetic polymer in orthopaedic application: A review. Mater. Today Proc..

[2] Ahmad A., Banat F., Alsafar H., Hasan S.W. (2022). An overview of biodegradable poly (lactic acid) production from fermentative lactic acid for biomedical and bioplastic applications. Biomass Convers. Biorefinery.

[3] Liu S., Wu G., Chen X., Zhang X., Yu J., Liu M., Zhang Y., Wang P. (2019). Degradation Behavior In Vitro of Carbon Nanotubes (CNTs)/Poly(Lactic Acid) (PLA) Composite Suture. Polymers.

[4] Ajmal S., Hashmi F.A., Imran I. (2022). Recent progress in development and applications of biomaterials. Mater. Today Proc..

[5] Hu X., Lin Z., He J., Zhou M., Yang S., Wang Y., Li K. (2022). Recent progress in 3D printing degradable polylactic acid-based bone repair scaffold for the application of cancellous bone defect. MedComm–Biomater. Appl..

[6] Jinnouchi H., Torii S., Sakamoto A., Kolodgie F.D., Virmani R., Finn A.V. (2019). Fully Bioresorbable Vascular Scaffolds: Lessons Learned and Future Directions. Nat. Rev. Cardiol..

[7] Toncheva A., Mincheva R., Kancheva M., Manolova N., Rashkov I., Dubois P., Markova N. (2016). Antibacterial PLA/PEG Electrospun Fibers: Comparative Study between Grafting and Blending PEG. Eur. Polym. J..

[8] Nedaipour F., Bagheri H., Mohammadi S. (2020). “Polylactic Acid-Polyethylene Glycol-Hydroxyapatite Composite” an Efficient Composition for Interference Screws. Nanocomposites.

[9] Sharma S., Singh A.A., Majumdar A., Butola B.S. (2019). Tailoring the Mechanical and Thermal Properties of Polylactic Acid-Based Bionanocomposite Films Using Halloysite Nanotubes and Polyethylene Glycol by Solvent Casting Process. J. Mater. Sci..

[10] Feng P., Jia J., Yu L., Min A., Yang S., Shuai C. (2021). Accelerated Degradation of Poly(L-Lactide) Bone Scaffold: Crystallinity and Hydrophilicity. Mater. Chem. Phys..

[11] Akrami M., Ghasemi I., Azizi H., Karrabi M., Seyedabadi M. (2016). A New Approach in Compatibilization of the Poly(Lactic Acid)/Thermoplastic Starch (PLA/TPS) Blends. Carbohydr. Polym..

[12] Bher A., Unalan I.U., Auras R., Rubino M., Schvezov C.E. (2019). Graphene Modifies the Biodegradation of Poly(Lactic Acid)-Thermoplastic Cassava Starch Reactive Blend Films. Polym. Degrad. Stabil..

[13] Momeni S., Rezvani Ghomi E., Shakiba M., Shafiei-Navid S., Abdouss M., Bigham A., Khosravi F., Ahmadi Z., Faraji M., Abdouss H. (2021). The Effect of Poly (Ethylene Glycol) Emulation on the Degradation of PLA/Starch Composites. Polymers.

[14] Yu M., Zheng Y., Tian J. (2020). Study on the Biodegradability of Modified Starch/Polylactic Acid (PLA) Composite Materials. RSC Adv..

[15] Watai J.S., Calvao P.S., Rigolin T.R., do Prado Bettini S.H., Souza A.M.C. (2020). Retardation Effect of Nanohydroxyapatite on the Hydrolytic Degradation of Poly (Lactic Acid). Polym. Eng. Sci..

[16] Naik A., Shepherd D.V., Shepherd J.H., Best S.M., Cameron R.E. (2017). The Effect of the Type of HA on the Degradation of PLGA/HA Composites. Mater. Sci. Eng. C-Mater. Biol. Appl..

[17] Patil T.V., Patel D.K., Dutta S.D., Ganguly K., Santra T.S., Lim K.-T. (2022). Nanocellulose, a Versatile Platform: From the Delivery of Active Molecules to Tissue Engineering Applications. Bioact. Mater..

[18] Iwatake A., Nogi M., Yano H. (2008). Cellulose Nanofiber-Reinforced Polylactic Acid. Compos. Sci. Technol..

[19] Abudula T., Saeed U., Memic A., Gauthaman K., Hussain M.A., Al-Turaif H. (2019). Electrospun Cellulose Nano Fibril Reinforced PLA/PBS Composite Scaffold for Vascular Tissue Engineering. J. Polym. Res..

[20] Benitez A.J., Walther A. (2017). Cellulose Nanofibril Nanopapers and Bioinspired Nanocomposites: A Review to Understand the Mechanical Property Space. J. Mater. Chem. A.

[21] Lundahl M.J., Klar V., Ajdary R., Norberg N., Ago M., Cunha A.G., Rojas O.J. (2018). Absorbent Filaments from Cellulose Nanofibril Hydrogels through Continuous Coaxial Wet Spinning. ACS Appl. Mater. Interfaces.

[22] Luo W., Cheng L., Yuan C., Wu Z., Yuan G., Hou M., Chen J.Y., Luo C., Li W. (2019). Preparation, Characterization and Evaluation of Cellulose Nanocrystal/Poly(Lactic Acid) in Situ Nanocomposite Scaffolds for Tissue Engineering. Int. J. Biol. Macromol..

[23] Shi Q., Zhou C., Yue Y., Guo W., Wu Y., Wu Q. (2012). Mechanical Properties and in Vitro Degradation of Electrospun Bio-Nanocomposite Mats from PLA and Cellulose Nanocrystals. Carbohydr. Polym..

[24] Shuai C., Yuan X., Yang W., Peng S., He C., Feng P., Qi F., Wang G. (2020). Cellulose Nanocrystals as Biobased Nucleation Agents in Poly-L-Lactide Scaffold: Crystallization Behavior and Mechanical Properties. Polym. Test.

[25] Zhou C., Shi Q., Guo W., Terrell L., Qureshi A.T., Hayes D.J., Wu Q. (2013). Electrospun Bio-Nanocomposite Scaffolds for Bone Tissue Engineering by Cellulose Nanocrystals Reinforcing Maleic Anhydride Grafted PLA. ACS Appl. Mater. Interfaces.

[26] Bitinis N., Fortunati E., Verdejo R., Bras J., Maria Kenny J., Torre L., Angel Lopez-Manchado M. (2013). Poly(Lactic Acid)/Natural Rubber/Cellulose Nanocrystal Bionanocomposites. Part II: Properties Evaluation. Carbohydr. Polym..

[27] Guerra A.J., Cano P., Rabionet M., Puig T., Ciurana J. (2018). 3D-Printed PCL/PLA Composite Stents: Towards a New Solution to Cardiovascular Problems. Materials.

[28] Pan M., Xu Z., Luo W., Yang Y., Teng T., Lin J., Huang H. (2021). In Vitro and in Vivo Properties Study of a Novel 3D-Printed Absorbable Pancreaticojejunostomy Device Made by Melting Blended Poly(p-Dioxanone)/Poly(Lactic Acid). Mater. Des..

[29] Alam F., Varadarajan K.M., Kumar S. (2020). 3D Printed Polylactic Acid Nanocomposite Scaffolds for Tissue Engineering Applications. Polym. Test.

[30] Zhu Q., Yu K., Li H., Zhang Q., Tu D. (2022). Rapid residual stress prediction and feedback control during fused deposition modeling of PLA. Int. J. Adv. Manuf. Technol..

[31] Khosravani M.R., Božić Ž., Zolfagharian A., Reinicke T. (2022). Failure analysis of 3D-printed PLA components: Impact of manufacturing defects and thermal ageing. Eng. Fail. Anal..

[32] Ueda T., Ishigami A., Thumsorn S., Kurose T., Kobayashi Y., Ito H. (2022). Structural, rheological, and mechanical properties of polyvinyl alcohol composites reinforced with cellulose nanofiber treated by ultrahigh-pressure homogenizer. Mater. Today Commun..

[33] Wang Q., Ji C., Sun L., Sun J., Liu J. (2020). Cellulose nanofibrils filled poly (lactic acid) biocomposite filament for FDM 3D printing. Molecules.

[34] (2012). Plastics-Determination of Tensile Properties-Part 2: Test Conditions for Moulding and Extrusion Plastics.

[35] Ambone T., Torris A., Shanmuganathan K. (2020). Enhancing the mechanical properties of 3D printed polylactic acid using nanocellulose. Polym. Eng. Sci..

[36] Tsuji H., Ikada Y. (2000). Properties and Morphology of Poly(l-Lactide) 4. Effects of Structural Parameters on Long-Term Hydrolysis of Poly(l-Lactide) in Phosphate-Buffered Solution. Polym. Degrad. Stab..

[37] Elsawy M.A., Kim K.-H., Park J.-W., Deep A. (2017). Hydrolytic Degradation of Polylactic Acid (PLA) and Its Composites. Renew. Sustain. Energy Rev..

[38] Siparsky G.L., Voorhees K.J., Miao F. (1998). Hydrolysis of Polylactic Acid (PLA) and Polycaprolactone (PCL) in Aqueous Acetonitrile Solutions: Autocatalysis. J. Environ. Polym. Degrad..

[39] Lyu S., Schley J., Loy B., Lind D., Hobot C., Sparer R., Untereker D. (2007). Kinetics and Time-Temperature Equivalence of Polymer Degradation. Biomacromolecules.

[40] (1999). Implants for surgery-Copolymers and blends based on polylactide-In vitro degradation testing.

[41] Balcerowiak W. (1998). The degree of crystallinity of polymer systems as determined by DSC. Polimery.

[42] Kalita N.K., Bhasney S.M., Kalamdhad A., Katiyar V. (2020). Biodegradable Kinetics and Behavior of Bio-Based Polyblends under Simulated Aerobic Composting Conditions. J. Environ. Manag..

[43] Piemonte V., Gironi F. (2013). Kinetics of Hydrolytic Degradation of PLA. J. Polym. Environ..

[44] Dimarzio E.A., Gibbs J.H. (1963). Molecular Interpretation of Glass Temperature Depression by Plasticizers. J. Polym. Sci. A Gen. Pap..

[45] Wang Q., Ji C., Sun J., Zhu Q., Liu J. (2020). Structure and properties of polylactic acid biocomposite films reinforced with cellulose nanofibrils. Molecules.

[46] Barrera D.A., Zylstra E., Lansbury P.T., Langer R. (1995). Copolymerization and degradation of poly (lactic acid-co-lysine). Macromolecules.

[47] Polak-Krasna K., Abaei A.R., Shirazi R.N., Parle E., Carroll O., Ronan W., Vaughan T.J. (2021). Physical and Mechanical Degradation Behaviour of Semi-Crystalline PLLA for Bioresorbable Stent Applications. J. Mech. Behav. Biomed. Mater..

[48] Sun X.C., Mazur M., Cheng C. (2023). A review of void reduction strategies in material extrusion-based additive manufacturing. Addit. Manuf..

